# Successful Bridging to Rituximab With Plasma Exchange in a Pediatric Patient With Severe Lupus Nephritis

**DOI:** 10.7759/cureus.102373

**Published:** 2026-01-27

**Authors:** Taro Yoshida, Hiroshi Sugahara, Keisuke Oikawa, Chinatsu Onodera, Manami Akasaka

**Affiliations:** 1 Pediatrics, Iwate Medical University, Morioka, JPN; 2 Pediatrics, Hachinohe Red Cross Hospital, Hachinohe, JPN; 3 Pediatrics, Iwate Prefectural Chubu Hospital, Kitakami, JPN; 4 Pediatrics, Iwate Medical University Hospital, Morioka, JPN

**Keywords:** acute kidney injury, immunosuppressive therapy in sle, lupus nephritis, pediatric, plasma exchange therapy, rituximab, steroid-sparing

## Abstract

We report a case of a 14-year-old girl diagnosed with class IV-G (A) lupus nephritis (LN), presenting with severe clinical manifestations, including nephrotic syndrome, acute kidney injury, and life-threatening hyperkalemia. The condition was managed with early initiation of plasma exchange (PE), followed by a combination of immunosuppressive therapies: high-dose corticosteroids, mycophenolate mofetil, rituximab, tacrolimus, and hydroxychloroquine. PE was promptly introduced after methylprednisolone pulse therapy to reduce circulating pathogenic autoantibodies and mitigate renal inflammation. Renal function gradually improved, accompanied by a marked decline in antibody levels. During recovery, the patient developed deep vein thrombosis and pulmonary embolism, which were managed with anticoagulation therapy. Belimumab was subsequently added to facilitate steroid tapering and maintain long-term disease control. Over an 18-month follow-up period, the patient achieved complete remission, with normalization of serologic markers and urinary findings, and corticosteroids were successfully discontinued. This case highlights the potential utility of early PE in enabling effective rituximab treatment for severe pediatric LN and underscores the importance of individualized, multimodal strategies to optimize outcomes in high-risk patients.

## Introduction

Lupus nephritis (LN) is a common and serious manifestation of systemic lupus erythematosus (SLE), particularly in the pediatric population, where it is associated with increased disease activity and worse renal outcomes compared to adults [1]. Despite advances in immunosuppressive therapy, if not promptly controlled, a subset of patients develops aggressive disease that progresses rapidly to end-stage kidney disease [2]. Early intervention and careful selection of therapeutic strategies are critical. Plasma exchange (PE) has been used selectively in severe complications of SLE, such as rapidly progressive glomerulonephritis or catastrophic antiphospholipid syndrome [3], but its broader role remains under investigation. In this report, we describe a pediatric patient with severe LN who was successfully treated with early PE and rituximab, highlighting the potential benefits of early aggressive intervention.

## Case presentation

A 14-year-old girl presented with a two-month history of facial rash and ear erythema, which progressed to generalized edema. Initial laboratory tests revealed thrombocytopenia (13.9 × 10⁴/μL), anemia (10.0 g/dL), hyperkalemia (6.6 mmol/L), elevated serum creatinine (0.97 mg/dL), hypoalbuminemia (1.5 g/dL), and nephrotic-range proteinuria (urinary protein/creatinine ratio: 6.6). Immunological evaluation demonstrated strongly positive antinuclear antibodies (ANA, 1:1,280) and marked hypocomplementemia (CH50 <10 U/mL, C3 27 mg/dL, and C4 2 mg/dL). Serological testing showed markedly elevated anti-dsDNA (380 U/mL) and anti-SSA (512.3 U/mL) antibody titers, with a Systemic Lupus Erythematosus Disease Activity Index (SLEDAI) score of 26. Additional baseline laboratory findings at presentation are summarized in Table 1.

**Table 1:** Baseline laboratory findings at presentation. WBC: white blood cell count; PT-INR: prothrombin time-international normalized ratio; APTT: activated partial thromboplastin time; Fbg: fibrinogen; FDP: fibrin/fibrinogen degradation products; BUN: blood urea nitrogen; Cre: creatinine; AST: aspartate aminotransferase; ALT: alanine aminotransferase; LD: lactate dehydrogenase; T-Bil: total bilirubin; CRP: C-reactive protein; IgG: immunoglobulin G; IgA: immunoglobulin A; IgM: immunoglobulin M; BNP: B-type natriuretic peptide; CH50: total hemolytic complement activity; C3: complement component 3; C4: complement component 4; ANA: antinuclear antibody; anti-ssDNA: anti-single-stranded DNA antibody; anti-dsDNA: anti-double-stranded DNA antibody; anti-RNP: anti-U1 ribonucleoprotein antibody; anti-Scl-70: anti-topoisomerase I antibody; anti-SS-A: anti-SSA/Ro antibody; anti-SS-B: anti-SSB/La antibody; anti-Sm: anti-Smith antibody; MMP3: matrix metalloproteinase-3; HPF: high-power field; U-NAG: urinary N-acetyl-β-D-glucosaminidase; β2MG: β2-microglobulin

Category	Parameter	Observed value	Reference range	Unit
Hematology	WBC	3,120	3,500–9,000	/µL
	Neutrophils	58.4	40–70	%
	Lymphocytes	25.6	20–45	%
	Monocytes	7.5	2–10	%
	Hemoglobin	10	11.5–15.5	g/dL
	Platelet count	13.9	15–35	×10⁴/µL
	Reticulocytes	3.5	2–8	×10⁴4/µL
Coagulation	PT-INR	0.85	0.9–1.1	-
	APTT	33.8	25–40	seconds
	Fibrinogen	389	200–400	mg/dL
	FDP	9	<5	µg/mL
	Antithrombin III	44	80–120	%
Biochemistry	Total protein	4.3	6.5–8.0	g/dL
	Albumin	1.5	3.8–5.3	g/dL
	BUN	31.9	7–20	mg/dL
	Cre	0.97	0.4–0.9	mg/dL
	AST	115	10–40	IU/L
	ALT	146	5–40	IU/L
	LD	268	120–240	IU/L
	T-Bil	<0.1	0.2–1.2	mg/dL
	Na	136	135–145	mmol/L
	K	6.6	3.5–5.0	mmol/L
	Cl	114	98–108	mmol/L
	Ca	7.6	8.8–10.2	mg/dL
	P (inorganic phosphate)	4.9	3.5–5.5	mg/dL
	CRP	<0.1	<0.3	mg/dL
Immunology	IgG	1209	700–1600	mg/dL
	IgA	217	70–400	mg/dL
	IgM	192	40–230	mg/dL
	BNP	17	<18	pg/mL
	Ferritin	843	10–150	ng/mL
	CH50	<10	30–45	U/mL
	C3	27	80–160	mg/dL
	C4	2	15–45	mg/dL
	Direct Coombs test	Positive	Negative	
Autoantibodies	ANA	1,280	Negative	Titer
	anti-ssDNA	>800	Negative	U/mL
	anti-dsDNA	380	Negative	U/mL
	anti-RNP	5.2	Negative	U/mL
	anti-Scl-70	<1	Negative	U/mL
	anti-SS-A	512.3	Negative	U/mL
	anti-SS-B	5.6	Negative	U/mL
	anti-Sm	<1.0	Negative	U/mL
	MMP3	30	<60	ng/mL
	Anticardiolipin IgG	<4.0	Negative	µg/mL
Urinalysis	Red blood cells	>100	0–4 /HPF	/HPF
	White blood cells	30–49	0–4 /HPF	/HPF
	Granular casts	1+	Negative	
	Epithelial casts	2+	Negative	
	Waxy casts	1+	Negative	
	Red blood cell casts	1+	Negative	
	Urine sodium	20	N/A	mEq/L
	Urine potassium	59.3	N/A	mEq/L
	Urine chloride	20	N/A	mEq/L
	Urine creatinine	177	N/A	mg/dL
	U-NAG	31.9	≤10	U/L
	U-Protein/Cre	6.4	<0.2	
	β2MG	<100	<300	µg/L

**Figure 1:**
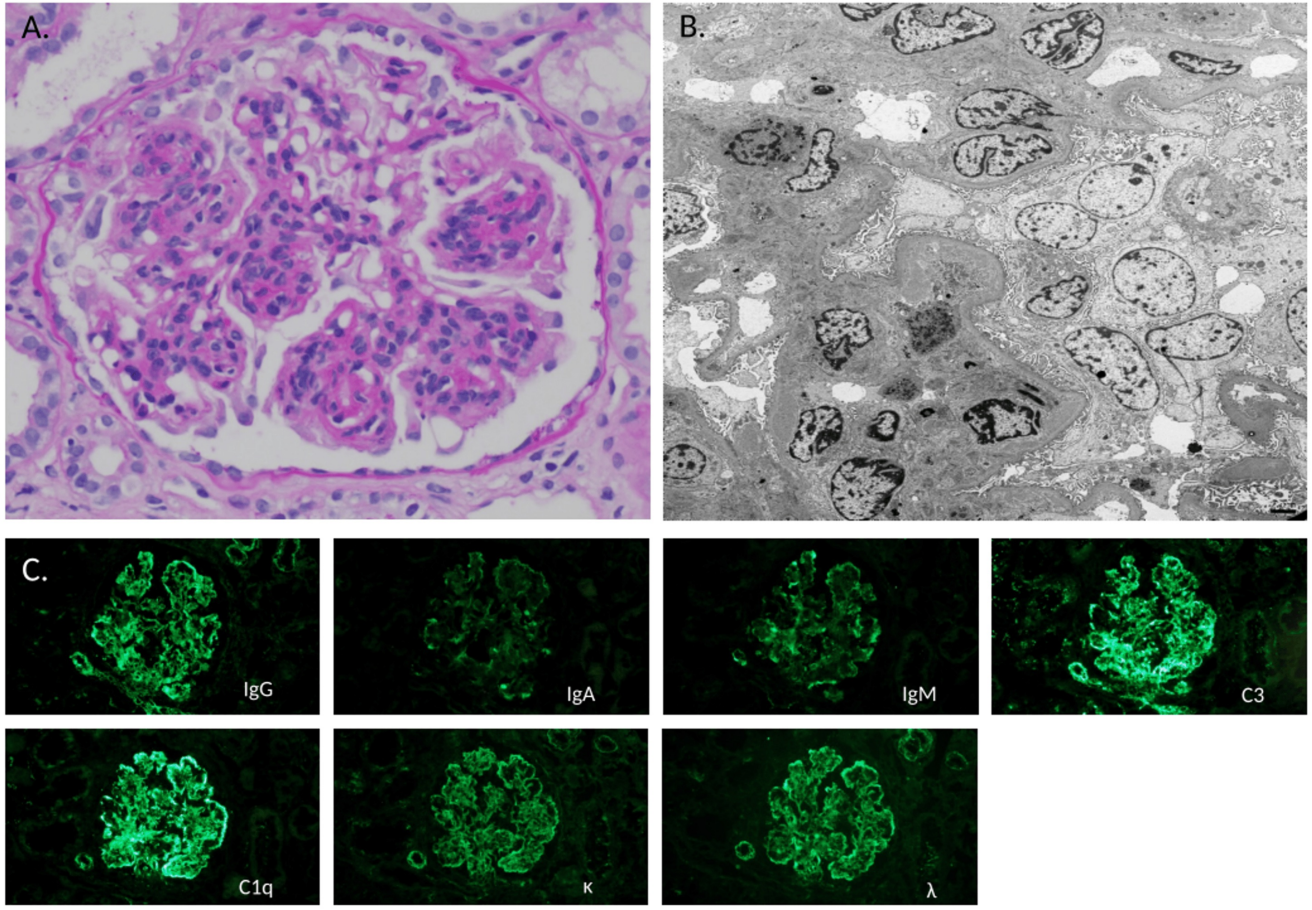
Histopathological findings of the renal biopsy specimens. (A) Mesangial proliferation is observed in the glomerulus (periodic acid-Schiff, 400×). (B) Transmission electron microscopy shows electron-dense deposits in the mesangial areas, as well as the subepithelial and subendothelial regions of the glomerular basement membrane (1,500×). (C) Immunofluorescence staining reveals positive reactivity along the glomerular capillary walls and in the mesangial areas across all the staining markers (200×).

A kidney biopsy was performed on hospital day 25. In the renal biopsy specimens, endocapillary hypercellularity was observed in 16 glomeruli (64%), mesangial cell proliferation in seven glomeruli (28%) (Figure 1a), intracapillary inflammatory infiltration in five glomeruli (20%), lobular appearance in three glomeruli (12%), and cellular crescents in two glomeruli (8%). A wire-loop lesion was found in one glomerulus (4%). Fibrinoid necrosis was not observed in all glomeruli. Interstitial fibrosis involved about 5% of the sampled cortex. Transmission electron microscopy revealed electron-dense deposits in the mesangial areas, as well as thesubepithelial and subendothelial areas of the glomerular basement membrane (Figure 1b). Immunofluorescence staining revealed positive reactivity along the glomerular capillary walls and in the mesangial areas in all staining markers (Figure 1c). Overall, renal biopsy findings were suggestive of diffuse LN: International Society of Nephrology/Renal Pathology Society 2018 modification: Class IV, Indices (Modified National Institutes of Health-NIH) of activity 8/24, and chronicity 2/12.

After correction of hyperkalemia, methylprednisolone pulse therapy was initiated on day two and administered for three cycles. Given the severity of LN, characterized by nephrotic syndrome, acute kidney injury with life-threatening hyperkalemia, extremely high anti-dsDNA titers, and marked systemic inflammation with a SLEDAI score of 26, rapidly progressive immune complex-mediated renal injury was strongly suspected. Despite steroid pulse therapy, the patient developed worsening fluid overload requiring intermittent hemodialysis. Hemodialysis was performed on hospital days 4, 5, 7, 10-12, and 18-19, and was discontinued after day 19 as the patient’s fluid balance, respiratory status, and renal function improved. PE was initiated on day four as a bridging strategy to promptly reduce circulating immune complexes and pathogenic autoantibodies until the therapeutic effects of subsequent immunosuppressive agents could be achieved. Mycophenolate mofetil was introduced on day eight. Although anemia and thrombocytopenia were present at admission, these cytopenias did not worsen after the initiation of methylprednisolone pulse therapy and supportive care. Although the histopathological results were not available at the time of treatment escalation, rituximab was administered on days 30 and 37 based on the severity of the clinical and serological findings, followed by the addition of hydroxychloroquine and tacrolimus. The subsequent biopsy results revealed diffuse LN with high activity, which supported the appropriateness of the chosen therapeutic approach. An angiotensin-converting enzyme inhibitor and an angiotensin II receptor blocker (ARB) were added for renal protection. Anti-dsDNA antibody levels declined rapidly after PE, and complement levels improved by day 11 and normalized by day 36. Belimumab was introduced on day 72. In accordance with the prescribing information, belimumab was administered at a dose of 200 mg per infusion, with the first three doses given at two-week intervals, followed by maintenance dosing every four weeks, adjusted according to disease activity. After sustained stabilization of clinical symptoms, renal function, and serological markers, mycophenolate mofetil was discontinued on hospital day 184, and tacrolimus was discontinued on hospital day 211.

**Figure 2:**
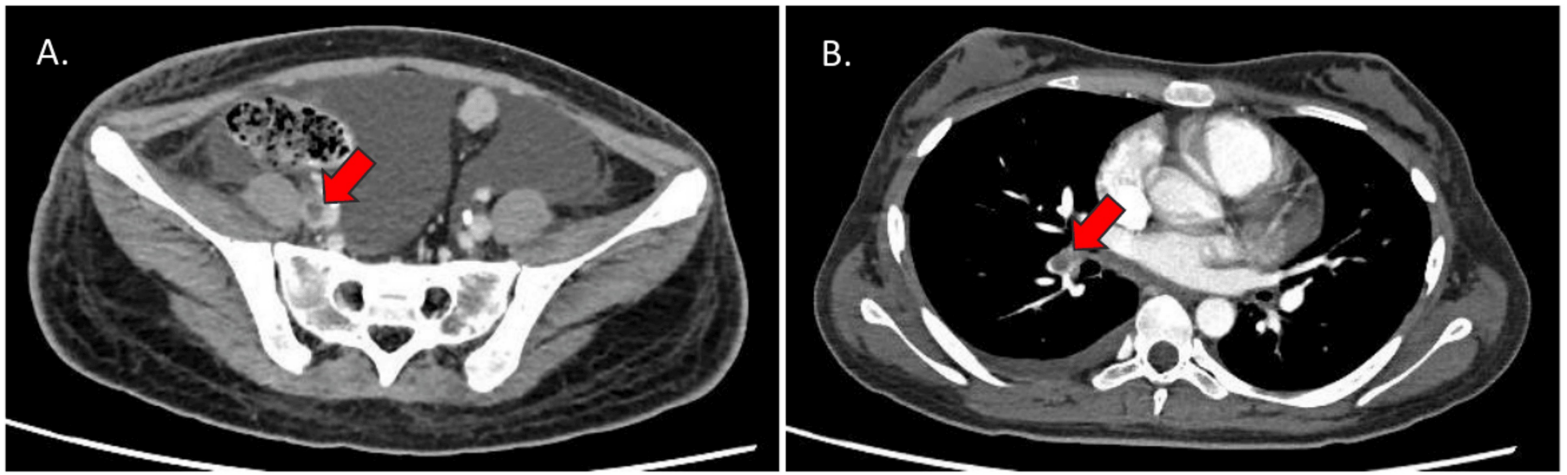
Contrast-enhanced CT findings of iliac vein thrombosis and pulmonary embolism. (A) Contrast-enhanced CT of the pelvis demonstrates thrombi in the right common iliac vein (arrows). (B) Contrast-enhanced CT of the chest reveals filling defects in the pulmonary arteries, consistent with pulmonary embolism (arrows).

On hospital day 42, the patient developed right lower leg edema and cough, prompting evaluation for thromboembolic complications. Contrast-enhanced CT revealed thrombi in the right common iliac vein and right external iliac vein, as well as a pulmonary artery embolism (Figure 2).

**Figure 3:**
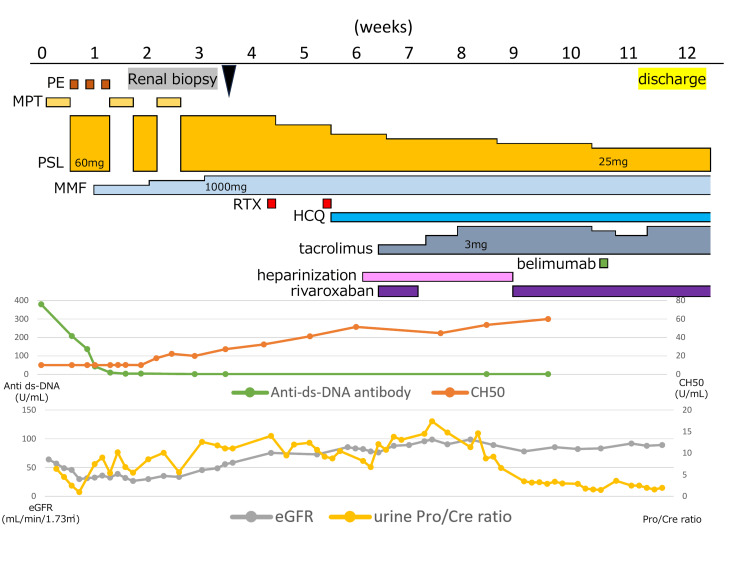
Clinical course of the patient. Alb: albumin; HCQ: hydroxychloroquine; MMF: mycophenolate mofetil; MPT: methylprednisolone pulse therapy; PE: plasma exchange; Pro/Cre: protein/creatinine; PSL: prednisolone; RTX: rituximab

Anticoagulation therapy with heparin followed by rivaroxaban was initiated. The common iliac vein thrombus resolved by day 72; however, a mural thrombus in the external iliac vein persisted, requiring continued anticoagulation. The patient was discharged on day 82 (Figure 3). Proteinuria improved by day 114, while urinary casts and hematuria resolved by days 309 and 526, respectively. Corticosteroids were tapered to 5 mg by day 365 and completely discontinued by day 555. At the last follow-up, the patient was maintained on hydroxychloroquine, belimumab, and an ARB alone, with a sustained SLEDAI score of 0.

## Discussion

This case illustrates the potential utility of early PE as a bridging therapy to rituximab in the management of severe pediatric LN. PE may rapidly reduce levels of pathogenic circulating immune complexes and autoantibodies, helping stabilize renal function during the critical early phase of treatment [4]. In our case, PE was initiated within the first week of admission, allowing timely rituximab administration and effective disease control. This strategy may be particularly beneficial in patients with impaired renal function, where immediate immunosuppression alone may be insufficient. Although the KDIGO 2024 guidelines recommend PE in cases of thrombotic microangiopathy or rapidly progressive glomerulonephritis in SLE [5], broader indications for its use remain unclear [6-8].

Given the patient’s young age, concerns regarding potential gonadotoxicity were substantial. Recent evidence has demonstrated that mycophenolate mofetil provides efficacy comparable to cyclophosphamide for induction therapy in LN, including pediatric populations. Therefore, we adopted a treatment strategy combining mycophenolate mofetil with early PE and rituximab to achieve rapid disease control while minimizing exposure to cyclophosphamide.

In recent years, the use of rituximab has increased in pediatric patients with refractory or severe LN, and favorable outcomes have been reported when rituximab is introduced in combination with PE as a bridging strategy [9,10]. Based on these considerations, rituximab was selected as an adjunctive therapy to provide effective immunosuppression during the acute phase without cyclophosphamide-associated toxicity.

A notable outcome in our case was the successful tapering and discontinuation of corticosteroids, facilitated by the use of rituximab and maintenance therapy with hydroxychloroquine and belimumab. Given the long-term side effects of corticosteroids, particularly in children, steroid-sparing strategies are of great clinical importance [11]. Although the use of belimumab in pediatric LN is still under evaluation [12,13], our experience suggests its potential role in maintaining remission and preventing disease flare.

Additionally, this case underscores the importance of thromboprophylaxis in pediatric SLE patients with central venous access and prolonged immobility. Risk factors for thrombosis during SLE treatment include antiphospholipid antibodies, LN, absence of thrombocytopenia, and corticosteroid use [14,15]. Despite the absence of antiphospholipid antibodies, our patient developed deep vein thrombosis and pulmonary embolism. In this case, physiotherapy was initiated on hospital day 16; however, prolonged bed rest was unavoidable due to the placement of a femoral central venous catheter for blood purification therapy. The catheter remained in place from hospital day 4 to day 23. An increasing trend in D-dimer levels was noted from day 21 and persisted after catheter removal, preceding the onset of pulmonary embolism symptoms on day 42. In addition to high-dose corticosteroid therapy, these factors likely contributed to the development of thromboembolic complications. Awareness of these risks and the implementation of early preventive strategies should be integral to the management of pediatric SLE patients.

Taken together, this case highlights the effectiveness of individualized, multimodal therapy, including early PE and targeted biologics, in managing severe pediatric LN. This study also highlights the need for standardized treatment protocols and further research to optimize therapeutic sequencing, improve outcomes, and reduce treatment-related toxicities in this vulnerable population.

## Conclusions

This case suggests that early PE may function as an effective bridging strategy to rituximab in pediatric patients with severe LN. Rapid removal of circulating immune complexes by PE appeared to facilitate disease stabilization and enable timely initiation of biologic therapy. Although the role of PE in LN remains controversial, this case highlights the importance of individualized, multimodal treatment approaches in high-risk pediatric patients.
